# Supplementary material for: Women’s traditional birth attendant utilization at birth and its associated factors in Angolella Tara, Ethiopia
Source: PLoS One. 2022 Nov 11;17(11):e0277504. doi: 10.1371/journal.pone.0277504 (PMC9651568; doi:10.1371/journal.pone.0277504)
Supplement: S1 File — (DOCX) [file pone.0277504.s001.docx]

**Questionnaire (English Version)**

The questionnaire is valid only for mothers who gave birth in the last two years.

For each question, make a circle around the number that corresponds to the answer; fill the blanks space with the answer.

Participant’s code number: ____________

**Part 1: Socio-demographic characteristics**

| S.No | Question | Response | Skip |
| --- | --- | --- | --- |
| 101 | How old are you? | ___________(completed years) |  |
| 102 | What is your marital status? | 1. Single 2. Married 3. Widowed 4. Divorced 5. Separated |  |
| 103 | Age at first marriage? | __________ |  |
| 104 | What is your level of education? | 1. Can’t read and write 2. able to read and write 3. Primary (1-8) 4. Secondary (9-12) 5. Diploma and above |  |
| 105 | Educational status of your partner? | 1. Can’t read and write 2. Able to read and write 3. Primary (1-8) 4. Secondary (9-12) 5. Diploma and above |  |
| 106 | What is your current occupation status? | 1. Housewife 2. Merchant 3. Daily laborer 4. Governmental employee 5. Private/NGO employee 6. Self-employed 7. student 8. Others (specify______) |  |
| 107 | Occupation of your partner? | 1. Farmer 2. Merchant 3. Daily laborer 4. Gov’t. employee 5. Private employee 6. Self-employed 7. student 8. Other specify_____ |  |
| 108 | How much is your family average household monthly income? | __________ ETB |  |
| 109 | Residence of the women? | 1. Rural 2. Urban |  |
| 110 | What is your religion? | 1. Orthodox Christian 2. Muslim 3. Protestant 4. Catholic 5. Others(specify______) |  |
| 111 | How many persons are living at home? | ____________ |  |
| 112 | Have you ever read newspapers? | 1. Yes 2. No |  |
| 113 | If yes, how often did you read? | 1. Every day 2. One time per week 3. Two times per week 4. Three and more |  |
| 114 | Have you ever heard radio? | 1. Yes 2. No |  |
| 115 | If yes, how often did you hear? | 1. Every day 2. One time per week 3. Two times per week 4. Three and more |  |
| 116 | How long does it take from your home to the nearby health facility on foot? | _____________ |  |

**Part II: Reproductive health service-related characteristics of women**

| **S. No** | **Questions** | | **Responses and coding** | **Skip** |
| --- | --- | --- | --- | --- |
| 201 | Have you ever used a family planning method? | | 1. Yes 2. No |  |
| 202 | Age at first pregnancy? | | ________ |  |
| 203 | How many times did you give birth (after 7 completed months) | | __________ |  |
| 204 | Total number of living children | | __________ |  |
| 205 | Do you have a history of medical illnesses during pregnancy? | | 1. Yes 2. No |  |
| 206 | Does the pregnancy planned | | 1. Yes 2. No |  |
| 207 | Do you have ANC visits during your last pregnancy? | | 1. Yes 2. No |  |
| 208 | Have you used TBAs services for your preceding birth (child)? | | 1. Yes 2. No |  |
| 209 | Who assisted your most recent delivery? | | 1. Skilled healthcare provider 2. HEW 3. TBA |  |
| 210 | Do you know about obstetric danger signs during pregnancy, childbirth, and postpartum period? | | 1. Yes 2. No |  |
| 211 | Have you ever faced obstetric danger signs during pregnancy, childbirth, and postpartum period? | | 1. Yes 2. No |  |
| 212 | Do you know about the presence of maternity waiting home in nearby HC. | | 1. Yes 2. No |  |
| 213 | Why do you prefer Traditional Birth attendants over skilled healthcare providers? | 1. They respect/ accept tradition 2. **Delay** of ambulance 3. No road access/infrastructure 4. It is the only maternity care that I know 5. They are existing in rural areas 6. Other specify | |  |
| 214 | The reason why not use HEWs/ skill health providers in the health institution? | 1. I cannot determine when to deliver 2. The labor was urgent 3. Cost of services 4. Lack of awareness 5. Health facility far away 6. Absence of privacy in health institutions. 7. Fear of operative procedures. 8. HEWs are not skilled. 9. Other specify | |  |
| 215 | Closest health facility  (multiple responses possible) | | 1. Health post 2. Private clinics 3. Hospital 4. Traditional healer |  |
| 216 | Major health facility use of women | | 1. Traditional healer 2. Health post 3. Private clinic 4. Hospital |  |
| 217 | Who decides the service providers for yourself? | | 1. Me alone 2. My husband alone 3. Both of us 4. Family members 5. Other specify |  |

**Part-III Attitude**

| **S. No** | **Questions**  1= Strongly disagree፣ 2=Disagree፣ 3=Neutral, 4= Agree, 5= Strongly agree, | **Responses and coding** | | | | |
| --- | --- | --- | --- | --- | --- | --- |
|  |  | **1** | **2** | **3** | **4** | **5** |
| 301 | Traditional birth attendants have adequate knowledge and skills to care for pregnant mother. |  |  |  |  |  |
| 302 | Hygiene practices of traditional birth attendants’ is adequate. |  |  |  |  |  |
| 303 | Child delivery process is easy, there is no need for skilled provider |  |  |  |  |  |
| 304 | Pregnancy related complications could not result from traditional birth attendants’ skill gaps. |  |  |  |  |  |
| 305 | Traditional birth attendants can treat birth-related problems. |  |  |  |  |  |
| 306 | Do you think to use traditional birth attendants for the next birth? |  |  |  |  |  |
| 307 | Modern health services are too expensive for women to afford |  |  |  |  |  |
| 308 | Delivery should not be at the health facility. |  |  |  |  |  |
| 309 | The presence of skilled birth attendants can’t reduce complications. |  |  |  |  |  |
| 310 | Traditional birth attendants give satisfied because it is user friendly care |  |  |  |  |  |
| 311 | Traditional birth attendants should continue to offer service |  |  |  |  |  |
| 312 | Traditional birth attendants services are effective |  |  |  |  |  |
| 313 | Traditional birth attendants give delivery services |  |  |  |  |  |
| 314 | Traditional birth attendants are patronized because we have no alternative. |  |  |  |  |  |
| 315 | Modern health care services are not available unlike  Traditional birth attendants. |  |  |  |  |  |

Thank you for your cooperation!
