# Supplementary material for: Women’s traditional birth attendant utilization at birth and its associated factors in Angolella Tara, Ethiopia
Source: PLoS One. 2022 Nov 11;17(11):e0277504. doi: 10.1371/journal.pone.0277504 (PMC9651568; doi:10.1371/journal.pone.0277504)
Supplement: Attachment — Submitted filename: PONE-D-22-02742_reviewed.pdf [file pone.0277504.s003.pdf]

PLOS ONE

Traditional birth attendant utilization at birth and associated factors among women who gave birth in the last two years preceding the survey in Angolella Tara District, Ethiopia

--Manuscript Draft--

**Manuscript Number:**	PONE-D-22-02742
**Article Type:**	Research Article
**Full Title:**	Traditional birth attendant utilization at birth and associated factors among women who gave birth in the last two years preceding the survey in Angolella Tara District, Ethiopia
**Short Title:**	Traditional birth attendant utilization at birth
**Corresponding Author:**	Birhan Tsegaw Taye<br>Debre Berhan University<br>Debrebirhan, Amhara regional state ETHIOPIA
**Keywords:**	Childbirth, Traditional birth attendants, Utilization, Prevalence, Ethiopia
**Abstract:**	Background<br>In low and middle-income countries, many childbirths still occur at home without the assistance of trained attendants. It is well known that unattended birth has been associated with serious life-threatening complications that will require appropriate and accessible care. On the other hand, 20-30% of neonatal mortality could be reduced by implementing skilled birth care services. This study aimed to assess reasons for traditional birth attendants' preference, utilization, and associated factors for women who gave birth in the last two yea rs in Angolella Tara District, Ethiopia.<br>Methods<br>A community-based cross-sectional study was employed among 416 women who gave birth in the last two years at rural Angolella Tara District. Study participants were recruited by using a simple random sampling technique. Data were collected using a structured, pretested, and interviewer-administered questionnaire. Epi Data 4.6 and SPSS version 25 were used for data entry and analysis, respectively. A multivariable logistic regression model was fitted to identify factors associated with women's utilization of traditional birth attendants. The level of significance in the last model was determined at a p-value of <0.05.<br>Result<br>In this study, 31.5% of the participants were used traditional birth attendants in their recent birth. Unmarried marital status (AOR 2.63; 95 % CI: 1.16, 5.97), age at first marriage (AOR 2.31; 95%CI: 1.30, 4.09), time to reach health facility (AOR= 3.46; 95% CI: 1.94, 6.17), know danger sign of pregnancy and childbirth (AOR=5.59, 95% CI; 2.89, 10.81), positive attitude towards traditional birth attendants (AOR=2.56 95% CI; 1.21,5.52), had antenatal care follow-up (AOR: 0.11 95% CI 0.058, 0.21), and listening radio (AOR=0.43; 95% CI: 0.18, 0.99) were significantly associated factors with the use of traditional birth attendants.<br><br>Conclusion<br>In this study, nearly one-third of women used traditional birth attendant services for their recent birth. Therefore, effort should be made by care providers and policymakers to ensure that modern health care services are accessible for women in a friendly and culturally sensitive manner. In addition, avocation through mass about the importance of maternal health service utilization, particularly antenatal care would be important.
**Order of Authors:**	Birhan Tsegaw Taye
	Mulualem Zerihun
	Tebabere Moltot Kitaw
	Tesfanesh Demisse
	Solomon Adanew Worku
	Girma Fitie

Powered by Editorial Manager® and ProduXion Manager® from Aries Systems Corporation

	Yeshinat Ambaw
	Nakachew Sewnet Amare
	Geremew Behulu
	Addisu Andualem Ferede
	Azmeraw Ambachew Kebede

Additional Information:	
**Question**	**Response**
**Financial Disclosure**<br><br>Enter a financial disclosure statement that describes the sources of funding for the work included in this submission. Review the submission guidelines for detailed requirements. View published research articles from *PLOS ONE* for specific examples.<br><br>This statement is required for submission and **will appear in the published article** if the submission is accepted. Please make sure it is accurate.<br><br>**Unfunded studies**<br>Enter: *The author(s) received no specific funding for this work.*<br><br>**Funded studies**<br>Enter a statement with the following details:<br>• Initials of the authors who received each award<br>• Grant numbers awarded to each author<br>• The full name of each funder<br>• URL of each funder website<br>• Did the sponsors or funders play any role in the study design, data collection and analysis, decision to publish, or preparation of the manuscript?<br>• **NO** - Include this sentence at the end of your statement: *The funders had no role in study design, data collection and analysis, decision to publish, or preparation of the manuscript.*<br>• **YES** - Specify the role(s) played.<br><br>* typeset	The author(s) received no specific funding for this work.
**Competing Interests**<br><br>Use the instructions below to enter a competing interest statement for this submission. On behalf of all authors,	The authors have declared that no competing interests exist.

disclose any competing interests that could be perceived to bias this work—acknowledging all financial support and any other relevant financial or non-financial competing interests.<br><br>This statement is required for submission and **will appear in the published article** if the submission is accepted. Please make sure it is accurate and that any funding sources listed in your Funding Information later in the submission form are also declared in your Financial Disclosure statement.<br><br>View published research articles from *PLOS ONE* for specific examples.<br><br>**NO authors have competing interests**<br><br>Enter: *The authors have declared that no competing interests exist.*<br><br>**Authors with competing interests**<br><br>Enter competing interest details beginning with this statement:<br><br>*I have read the journal's policy and the authors of this manuscript have the following competing interests: [insert competing interests here]*<br><br>* typeset	
**Ethics Statement**<br><br>Enter an ethics statement for this submission. This statement is required if the study involved:<br><br>• Human participants<br>• Human specimens or tissue<br>• Vertebrate animals or cephalopods<br>• Vertebrate embryos or tissues<br>• Field research<br><br>Write "N/A" if the submission does not require an ethics statement.	Debre Berhan University ethics committee

General guidance is provided below. Consult the submission guidelines for detailed instructions. **Make sure that all information entered here is included in the Methods section of the manuscript.**<br><br>**Format for specific study types**<br><br>**Human Subject Research (involving human participants and/or tissue)**<br>• Give the name of the institutional review board or ethics committee that approved the study<br>• Include the approval number and/or a statement indicating approval of this research<br>• Indicate the form of consent obtained (written/oral) or the reason that consent was not obtained (e.g. the data were analyzed anonymously)<br><br>**Animal Research (involving vertebrate animals, embryos or tissues)**<br>• Provide the name of the Institutional Animal Care and Use Committee (IACUC) or other relevant ethics board that reviewed the study protocol, and indicate whether they approved this research or granted a formal waiver of ethical approval<br>• Include an approval number if one was obtained<br>• If the study involved *non-human primates*, add *additional details* about animal welfare and steps taken to ameliorate suffering<br>• If anesthesia, euthanasia, or any kind of animal sacrifice is part of the study, include briefly which substances and/or methods were applied<br><br>**Field Research**<br><br>Include the following details if this study involves the collection of plant, animal, or other materials from a natural setting:<br>• Field permit number<br>• Name of the institution or relevant body that granted permission	
**Data Availability**<br><br>Authors are required to make all data	Yes - all data are fully available without restriction

underlying the findings described fully available, without restriction, and from the time of publication. PLOS allows rare exceptions to address legal and ethical concerns. See the PLOS Data Policy and FAQ for detailed information.<br><br>A Data Availability Statement describing where the data can be found is required at submission. Your answers to this question constitute the Data Availability Statement and **will be published in the article**, if accepted.<br><br>**Important:** Stating 'data available on request from the author' is not sufficient. If your data are only available upon request, select 'No' for the first question and explain your exceptional situation in the text box.<br><br>Do the authors confirm that all data underlying the findings described in their manuscript are fully available without restriction?	
**Describe where the data may be found in full sentences. If you are copying our sample text, replace any instances of XXX with the appropriate details.**<br><br>• If the data are **held or will be held in a public repository**, include URLs, accession numbers or DOIs. If this information will only be available after acceptance, indicate this by ticking the box below. For example: *All XXX files are available from the XXX database (accession number(s) XXX, XXX.*).<br>• If the data are all contained **within the manuscript and/or Supporting Information files**, enter the following: *All relevant data are within the manuscript and its Supporting Information files.*<br>• If neither of these applies but you are able to provide **details of access elsewhere**, with or without limitations, please do so. For example:<br><br>*Data cannot be shared publicly because of [XXX]. Data are available from the*	All relevant data are within the manuscript and its Supporting Information files.

XXX Institutional Data Access / Ethics Committee (contact via XXX) for researchers who meet the criteria for access to confidential data.<br><br>*The data underlying the results presented in the study are available from (include the name of the third party and contact information or URL).*<br>• This text is appropriate if the data are owned by a third party and authors do not have permission to share the data.<br><br>* typeset	
Additional data availability information:	Tick here if the URLs/accession numbers/DOIs will be available only after acceptance of the manuscript for publication so that we can ensure their inclusion before publication.

Powered by Editorial Manager® and ProduXion Manager® from Aries Systems Corporation

Manuscript Click here to access/download;Manuscript;Manuscript.docx

Research article

Traditional birth attendant utilization at birth and associated factors among women who gave birth in the last two years preceding the survey in Angolella Tara District, Ethiopia

Birhan Tsegaw Taye*[1], Mulualem Silesh Zerihun[1], Tebabere Moltot Kitaw[1], Tesfanesh Lemma Demisse[1], Solomon Adanew Worku[1], Girma Wogie Fitie[1], Yeshinat Lakew Ambaw[1], Nakachew Sewnet Amare[1], Geremew Kindie Behulu[1], Addisu Andualem Ferede[2], and Azmeraw Ambachew Kebede[3]

[1] School of Nursing and Midwifery, Asrat Woldeyes Health Science Campus, Debre Berhan University, Debre Berhan, Ethiopia.

[2] Department of Midwifery, College of Medicine and Health Science, Debre Markos University, Debre Markos, Ethiopia.

[3] Department of Clinical Midwifery, School of Midwifery, College of Medicine and Health Sciences, University of Gondar, Gondar, Ethiopia.

*tsegawbirhan2@gmail.com

ABSTRACT

Background

In low and middle-income countries, many childbirths still occur at home without the assistance of trained attendants. It is well known that unattended birth has been associated with serious life-threatening complications that will require appropriate and accessible care. On the other hand, 20-30% of neonatal mortality could be reduced by implementing skilled birth care services. This study aimed to assess reasons for traditional birth attendants' preference, utilization, and associated factors for women who gave birth in the last two years in Angolella Tara District, Ethiopia.

Methods

A community-based cross-sectional study was employed among 416 women who gave birth in the last two years at rural Angolella Tara District. Study participants were recruited by using a simple random sampling technique. Data were collected using a structured, pretested, and interviewer-administered questionnaire. Epi Data 4.6 and SPSS version 25 were used for data entry and analysis, respectively. A multivariable logistic regression model was fitted to identify factors associated with women's utilization of traditional birth attendants. The level of significance in the last model was determined at a p-value of <0.05.

Result

In this study, 31.5% of the participants were used traditional birth attendants in their recent birth. Unmarried marital status (AOR 2.63; 95 % CI: 1.16, 5.97), age at first marriage (AOR 2.31; 95%CI: 1.30, 4.09), time to reach health facility (AOR= 3.46; 95% CI: 1.94, 6.17), know danger sign of pregnancy and childbirth (AOR=5.59, 95% CI; 2.89, 10.81), positive attitude towards traditional birth attendants (AOR=2.56 95% CI; 1.21,5.52), had antenatal care follow-up (AOR: 0.11 95% CI 0.058, 0.21), and listening radio (AOR=0.43; 95% CI: 0.18, 0.99) were significantly associated factors with the use of traditional birth attendants.

Conclusion

In this study, nearly one-third of women used traditional birth attendant services for their recent birth. Therefore, effort should be made by care providers and policymakers to ensure that modern health care services are accessible for women in a friendly and culturally sensitive manner. In addition, avocation through mass about the importance of maternal health service utilization, particularly antenatal care would be important.

Keywords: Childbirth, Traditional birth attendants, Utilization, Prevalence, Ethiopia

Introduction

The World Health Organization (WHO) defines a traditional birth attendant (TBAs) as a person who assists the mother during childbirth, and who initially acquired her skills by delivering babies herself or through an apprenticeship with other TBAs (1,2). TBAs are involved in 60–80% of all deliveries in rural regions of developing countries (3).

Globally, there is a target to reduce the maternal mortality ratio (MMR) to less than 70/100, 000 live births and no country should have MMR greater than 140/100, 000 live births by 2030 (4). As a WHO report, approximately 810 women died every day in 2017 as a result of pregnancy and childbirth complications; with 94% of them living in low and lower-middle-income countries (5). In Ethiopia, the MMR was 401 per 100,000 live births in 2019, one of the highest in the world (HSTP-II) (6). The large majority of these maternal deaths occur as a result of unavailable, inaccessible, costly, or poor quality maternal health care services (7–9)

Skilled care during the maternal continuum of care can save the lives of women and newborns (5,10). Improving access to skilled care during pregnancy, childbirth, and the postpartum period is a top priority to improve maternal health and meet the sustainable development goals (SDG3) (11). Since the launching of the Health Extension Program in 2003, there has been a change in Ethiopian policy that TBAs are no longer allowed to attend births (12). However, most women in developing countries give birth outside of health facilities with the help of TBAs and their lifetime risk of pregnancy-related death is higher (5,9). In 2017, the MMR in low-income nations was 462/100,000 live births, compared with 11/100,000 live births in high-income countries (5). Although women believed that that complication might arise from TBA care (13), low maternal educational level, rural residence, poor family wealth index,

unemployed status, having >5 living children, married, predominantly farmers, married to spouses who were farmers without formal education, user-friendly care from TBAs, easily accessible, delivery environment familiar to the clients' respect from TBAs for the religious beliefs of clients were significant factors for women to used TBAs (9,13–18).

Ethiopian mini demographic health survey (EMDHS), 2019 report showed that only 50% of births were attended by skilled birth attendants, nevertheless, the Ethiopian government planned to increase the proportion of skilled birth attendants to 90% by 2020 (6). Hence, home birth rates are much higher in rural parts of the country than in urban areas (urban: 29.6% vs. rural: 60%) (19) Also, a significant proportion of women was not still engaged fully in the continuum of maternal care with significant dropouts. For instance, 74% of pregnant women undergo at least one antenatal care (ANC) visit, 48% of women gave birth in the health institution, and 34% of women received postnatal care in the first 2 days after birth (19). Though there is a need to improve the utilization of health facility deliveries with skilled birth attendants, still many women gave birth at home with the aid of TBAs in rural and deprived communities of Ethiopia. In this regard, women's attitude towards TBAs and reasons for preference for TBAs were lacking in previous research. Therefore, this study aimed to assess reasons for traditional birth attendants' preference, utilization, and associated factors among women who gave birth in the last two years in Angolella Tara District, Ethiopia.

Research questions:

1. To what extent do women utilize on TBAs?

2. What are the potential factors affecting the utilization of TBAs for child delivery services?

3. Why do women prefer TBAs?

Methods and materials

Study design, period, and setting

A community-based cross-sectional study was conducted from June 1st to 15th, 2021. This study was conducted in rural Angolella-Tara district, Amhara regional state, Northeast Ethiopia. The district is located at the eastern edge of the Ethiopian highlands in the North Shewa zone, 112 km Northeast of Addis Ababa (the capital city of Ethiopia). It is called in part after one of the capitals of the former principality of Shewa, Angolella Tara district. Currently, the district has 21 kebeles with an area of 782.49 km^2 and a total of an estimated population of 82,349, of whom 40,500 were women (based on the 2007 national census). Also, there are four health centers and 21 health posts serving the community.

Source population and study population

All women who gave birth in the last two years at Angolella-Tara district were the source populations. All women who gave birth in the last two years in selected kebeles at Angolella -Tara district during the data collection period were the study population.

Sample size determination

The sample size for this study was determined by using a single proportion formula by considering the following assumptions: a 50% proportion of women's utilization of TBAs, 95% level of confidence, and 5% margin of error.

$$n = \frac{Z\alpha/2)^2 p(1-p)}{d^2} \text{Therefore}, n = \frac{(1.96)^{2*} 0.5(1-0.5)}{(0.05)2} = 384$$

By considering a 10% non-response rate, the minimum adequate sample size was 422.

Sampling technique and procedure

From the total of 21 kebeles, nine kebeles were selected randomly using a lottery method. . The list of the study participants was gained from health extension workers (HEWs) and local administrators. The sampling frame was designed by numbering the list of women. Then, the total sample size was distributed to each selected "kebeles" proportional to the size. Lastly, the eligible women were chosen by a simple random sampling technique.

Variables of the study

Dependent variable

Utilization of TBAs as birth attendants (Utilized/not Utilized).

Independent variables

Socio-demographic characteristics (Maternal age, Maternal occupation, Husband occupation, Mother's educational status, Husband educational status, Media exposure, Income, Marital status, Family size, listening radio, read newspaper).

Reproductive health-related characteristics (Parity, Family planning, planned pregnancy, Assistant of the recent delivery, Knowledge on obstetric danger signs, having ANC visit, Family size, Alive children, Healthcare service decision making, know maternity waiting home); and Attitude towards TBAs**.**

Measurement and operational definitions

Traditional birth attendant: A TBA is defined by the WHO as a person, generally a woman, who assists pregnant women during childbirth and who learned the skills by attending homebirths, potentially with the help of other TBAs (1,20).

Skilled attendant: is a professionally trained healthcare provider having the essential skills to manage normal labor and delivery, recognize complications early and perform any essential interventions including early referral (21).

Utilization of traditional birth attendants: when women delivered at home or birth that takes place in a residence without a skilled birth attendant; the possible answers were Yes or No. A score of "1" was given for Yes and a score of "0" was given for No which was dichotomized as utilized and not utilized (2,12).

Women's attitude - Women's attitude towards TBAs was measured using 15 questions: Each question has five points Likert scale (1= strongly disagree, 2= disagree, 3 = neutral, 4= agree, 5= strongly agree). The total score was 15–75 and women who scored 50% and above value were considered as having a positive attitude and those who scored below 50% were considered as a negative attitude.

Know obstetric danger signs: women who were able to list at least two of the key danger signs (vaginal bleeding, swelling of face, fingers, severe persistent abdominal pain, blurring of vision, severe recurrent frontal headache, high-grade fever, and swelling of the face) categorized know the danger signs and no otherwise (22).

Planned pregnancy: A woman who plans to become pregnant by making lifestyle choices for optimal health in advance of the planned conception.

Maternity waiting home: If mothers are living far from the delivery center, they shall be admitted to a maternity waiting home (residential facility) which is located near or within hospitals in their final weeks of pregnancy to bridge the geographical gap in obstetric care (23).

Data collection tools and quality control

Data were collected using a pre-tested, structured, and interviewer-administered questionnaire through face-to-face interviews. The questionnaire was prepared by reviewing related literature (references). The questionnaire was first prepared in English and translated into Amharic (local language), and then back to English to maintain the consistency of the questionnaire. Six Bachelor of Science and one MSc midwives were recruited for data collection and supervision, respectively. Before the actual data collection, a pretest was done at Baso district on 5% of the sample size out of the study sites to check language clarity, and appropriateness of the questionnaire based on the pretest, necessary amendments were done.

Data processing and analysis

All information was recorded corresponding to the code given to each respondent. Data were checked, coded, and entered into EpiData version 4.6, and was exported to SPSS version 25 for analysis. Descriptive statistics were used to present the participants' characteristics. Bivariable and multivariable logistic regressions analysis were employed. Variables with a P value of less than 0.25 on bivariate logistic regression were selected as a candidate for multivariate logistic regression to identify statistically significant factors and the level of significance was determined at a p-value of <0.05 and the strength of association was interpreted using the adjusted odds ratio (AOR) with its 95% confidence interval (CI).

Ethical consideration

The study was conducted under the Ethiopian Health Research Ethics Guideline and the declaration of Helsinki. Ethical clearance was obtained from Debre Berhan University ethical committee. A formal letter of administrative approval was gained from the Angolella Tara district and local administrator. Written informed consent was obtained from respondents before data collection by explaining the purpose of the study. The name of participants was not written and confidentiality was maintained throughout the study. The data collector was explained that the respondent can withdraw from the study that participation was voluntary.

Result

Sociodemographic characteristics

A total of 416 women were participated in this study, giving a response rate of 98.58%. Of these 213 (51.2%) were in the age group of 26-30 and the mean age of the study participants was 33.56 years (SD ±8.01). The majority (86.8%) of the study participants were married and almost all (99.3%) of them were Orthodox Christian by religion (Table1).

Reproductive health service-related characteristics of women

More than half of women (53.4%) used family planning methods. About 76.9% of the pregnancies were unplanned. Two hundred sixty (62%) of women were assisted by a skilled birth attendant for their most recent birth. Moreover, 299 (71.9%) of the respondents had ANC follow-up in the preceding pregnancy (71.4%) (Table 2).

Utilization and attitude of women towards TBAs

Overall, 131(31.5%; 95% CI: 27%, 36%) of women used TBAs services for their most recent childbirth. More than half of women (58.2%) have a negative attitude towards TBAs.

Women’s reason for preference of traditional birth attendants

There are many reasons for women’s preference for traditional birth attendants. Among these, TBAs are available in rural areas (36.54%), roads access problems (19.47%), respect of culture and tradition (16.35%), they know only TBAs (14.90%), and unavailability and delay of ambulance upon call (12.74%) (Figure 1).

Factors associated with women's utilization of traditional birth attendants

Multivariable logistic regression analysis revealed that being unmarried, age at first marriage, ANC follow up, time to reach a health facility, listening to radio, didn't know obstetric danger signs, and positive attitude towards TBAs were factors associated with utilization of TBAs by women.

Accordingly, this study found that those women with unmarried current marital relations were 2.63 (AOR = 2.63; 95 % CI: 1.16, 5.97) times more likely to use TBAs as compared to married women. Those mothers whose age at first marriage was less than 18 year-olds were 2.31 (AOR = 2.31; 1.30, 4.09) times more likely to use TBA services. This study also established that women who traveled for greater than one hour were 3.46 times more likely to have TBA as compared with those women who traveled for less than an hour (AOR = 3.46; 95% CI: 1.94, 6.17). Mothers who didn't know danger signs of pregnancy and childbirth were 5.59 times more likely to utilize TBAs than their counterparts (AOR = 5.59; 95% CI: 2.89, 10.81).

Moreover, mothers who have a positive attitude towards TBAs were 2.23 times more likely to use TBAs than those who have negative attitude AOR = 2.56; 95% CI: 1.21, 5.52). However, women who have ANC follow-up were 89% less likely to use TBA services during birth. Likewise, participants who listen to the radio at least once per week were 57% less likely utilizing TBAs for their childbirths as compared to their counterparts (AOR = 0.43; 95% CI: 0.18, 0.99) (Table 3).

Discussion

The key to reducing MMR and improving maternal health is increasing proper utilization of maternal health services by skilled health personnel throughout pregnancy and childbirth. However, health facility delivery is significantly lower in Ethiopia. In recent years, there has been increasing debate over the usefulness of TBAs in maternal health care. Opponents of TBA care are of the view that TBAs have done little to improve maternal health. After the advent of the World Health Organization's Safe Motherhood initiative, the enthusiasm turned away from TBAs (24) as they are unskilled and unable to prevent or treat the complications during pregnancy or childbirth that leads to maternal deaths (25). Thus, the finding of this study showed that 31.5% of women gave birth by TBAs in their current birth which is lower

than the study done in Nigeria; 65% of the women had been delivered by TBAs (26). The possible explanation could be the Nigerian government encourages the integration of TBAs into the health sector for improving maternal health (27).

The current study found that time taken to the nearby health facility was significantly associated with the use of TBAs for childbirth. It has been shown that women who travel for greater than an hour to reach health facilities were 3.46 times high likely to attend their birth by TBAs as compared to those women traveling for less than one hour. This finding is consistent with studies conducted in Ethiopia (28,29), Eretria (30), Kenya (31), and Zambia (32). This could be because the distance to a health facility is one of the well-known barriers to healthcare access especially in developing countries (33,34).

The present study also showed that having ANC follow-up had a significant association with the use of TBA services. Thus, women who have ANC follow-up for their recent pregnancy were 89% less likely assisted by TBAs. This finding is supported by the study conducted in Kenya, in which women who have no ANC follow-up pose an increased utilization of TBAs (35). This could be justified by ANC utilization increasing the use of skilled birth attendants and institutional delivery.

Women’s at first marriage was a significant risk factor for TBA utilization. In this regard, women who were married before or at the age of 18-year-old were 2.31 times more likely to use TBA in their current childbirth as compared to their older counterparts. This finding is in agreement with studies conducted in Kenya (31), and Zambia (32). This could be because; experience in pregnancy and pregnancy-related complications increases as women’s age advances which could help them to visit health facilities thereby obtaining a comprehensive and favorable awareness on maternal health services in the subsequent pregnancy. Besides, older women could have a higher decision-making power regarding their health and health care utilization as compared to young age women (36,37).

The current study also revealed that the odds of having TBA utilization among women who had a positive attitude were 2.23 times higher as compared to those women who had a negative attitude towards TBAs. This finding is consistent with a previous study conducted in Ethiopia (38). Thus, significant numbers of maternal and neonatal mortality occurred without ever reaching a health facility or due to delays in seeking care in Ethiopia. Hence, interventions that

will address health education for women including husbands/spouses, and other family members would be helpful to enhance the utilization of SBAs.

Moreover, this finding indicated that those women who didn't know obstetric danger signs were 5.59 times more likely to use TBAs for their childbirth. This suggests that complications during previous pregnancy and childbirth make women aware of the obstetric danger signs and the benefits of SBAs (39). Consequently, they could get information regarding the danger signs of pregnancy and the importance of utilizing SBAs for subsequent pregnancies.

The odds of TBA service utilization among women who listen to radio at least once per week were less likely to approve TBAs services utilization. The possible explanation could be due to the reality that information, education, and communication regarding maternal, neonatal, and child health improvement could be disseminated through different mass media (40,41).

Importantly, this study examined the reasons for the preference of the TBAs over SBAs. Thus, the major reasons why women prefer TBAs other than SBAs are: TBAs are available in a rural area, roads access problems, TBAs respect culture and tradition, know only TBAs and there is a delay of an ambulance. This finding is supplemented by a study conducted from Ethiopia, lack of respectful care at health care facilities and the friendliness of traditional birth attendants encourage TBA services (42). Even in the presence of health professionals and HEWs, TBAs are the first choice of women in the rural community as services are provided within the home and involved in TBAs tailored to the community's norm such as burying placenta inside the home (14). This could be the reason why most women in rural communities build trust in TBAs. Lack of trust in the HEWs' capacity and limited coordination TBAs, HEWs, and professional healthcare providers contribute to TBAs being the choice of birth attendants (43–45).

This finding calls policymakers like the Ethiopian Ministry of Health (MoH) and other stakeholders should strengthen health extension programs based on the assumption that access to and quality of primary health care in rural communities can be improved through the transfer of health knowledge, attitude, and skills to households.

Limitations of the study

We are pleased to acknowledge some of the limitations of the current study. First, as the area is not well studied, we didn't find adequate studies to compare and contrast our findings with others, which made our discussion shallow. Second, due to the cross-sectional nature of the study design, the cause-effect relationship between the outcome and explanatory variables might be impossible. Third, it is impossible to couch the validity of the responses that social desirability bias may be introduced. Despite these limitations, our findings provide valuable information about women's TBA utilization, factors associated with it, and reasons for preference for TBA services.

Conclusion

The extent of TBA service utilization among women who gave birth in the last two years was high in the study setting. Community-based interventions like health education on obstetric danger signs by giving more emphasis on the benefits of using ANC follow up is the midst for the reduction of TBA service utilizations. Besides, strengthening access to transportation, health information communication mass media will enhance the use of SBAs. Exploring barriers and facilitators of deep-rooted socio-cultural and behavioral factors in this sub-theme would be helpful. In this regard, strengthening respectful maternity care service throughout the continuum of care could help to increase women's preference towards SBAs.

Supporting information

S1 file: English version of the questionnaire.

S2 file: SPSS dataset.

Acknowledgments

We appreciate and wish to thank Debre Berhan University for providing ethical clearance to carry out this study. We also would like to acknowledge the Woreda administrators, data collectors, and study participants.

Authors' Contributions

Conceptualization: Birhan Tsegaw Taye

Data curation: Birhan Tsegaw Taye, Mulualem Silesh Zerihun, Tebabere Moltot Kitaw, Tesfanesh Lemma Demisse, Solomon Adanew Worku, Girma Wogie Fitie, Yeshinat Lakew Ambaw, Nakachew Sewnet Amare, Geremew Kindie Behulu, Addisu Andualem Ferede, and Azmeraw Ambachew Kebede.

Formal analysis: Birhan Tsegaw Taye, Mulualem Silesh Zerihun, Tebabere Moltot Kitaw, Tesfanesh Lemma Demisse, Solomon Adanew Worku, Girma Wogie Fitie, Yeshinat Lakew Ambaw, Nakachew Sewnet Amare, Geremew Kindie Behulu, Addisu Andualem Ferede, and Azmeraw Ambachew Kebede.

Investigation: Birhan Tsegaw Taye, Mulualem Silesh Zerihun, Tebabere Moltot Kitaw, Tesfanesh Lemma Demisse, Solomon Adanew Worku, Girma Wogie Fitie, Yeshinat Lakew Ambaw, Nakachew Sewnet Amare, Geremew Kindie Behulu, Addisu Andualem Ferede, and Azmeraw Ambachew Kebede.

Methodology: Birhan Tsegaw Taye, Mulualem Silesh Zerihun, Tebabere Moltot Kitaw, Tesfanesh Lemma Demisse, Solomon Adanew Worku, Girma Wogie Fitie, Yeshinat Lakew Ambaw, Nakachew Sewnet Amare, Geremew Kindie Behulu, Addisu Andualem Ferede, and Azmeraw Ambachew Kebede.

Validation: Birhan Tsegaw Taye, Mulualem Silesh Zerihun, Tebabere Moltot Kitaw, Tesfanesh Lemma Demisse, Solomon Adanew Worku, Girma Wogie Fitie, Yeshinat Lakew Ambaw, Nakachew Sewnet Amare, Geremew Kindie Behulu, Addisu Andualem Ferede, and Azmeraw Ambachew Kebede.

Visualization: Birhan Tsegaw Taye, Mulualem Silesh Zerihun, Tebabere Moltot Kitaw, Tesfanesh Lemma Demisse, Solomon Adanew Worku, Girma Wogie Fitie, Yeshinat Lakew Ambaw, Nakachew Sewnet Amare, Geremew Kindie Behulu, Addisu Andualem Ferede, and Azmeraw Ambachew Kebede.

Writing-original draft: Birhan Tsegaw Taye

Writing- review and editing: Birhan Tsegaw Taye, Mulualem Silesh Zerihun, Tebabere Moltot Kitaw, Tesfanesh Lemma Demisse, Solomon Adanew Worku, Girma Wogie Fitie,

Yeshinat Lakew Ambaw, Nakachew Sewnet Amare, Geremew Kindie Behulu, Addisu Andualem Ferede, and Azmeraw Ambachew Kebede.

Data Availability Statement

The datasets collected and analyzed for the current study are available within the manuscript and its supporting information files.

Funding

The authors received no specific funds for this work.

Competing interests

The authors declare that they have no conflict of interest in this work.

Abbreviations

ANC: Antenatal Care, **MMR**: Maternal Mortality Ratio, **MOH**: Ministry of Health: **MWH**: Maternity Waiting Homes, **PPH**: Post-Partum Hemorrhage, **SBA**: Skilled birth attendant, **SDG**: Sustainable Development Goal: **TBA**: Traditional Birth Attendance, **WHO**: World Health Organization

Table 1: Socio-Demographic Characteristics of women in Angolella Tara district northeast Ethiopia, 2021 (n= 416)

Characteristics	Frequency	Percentage (%)
**Age**		
≤25	29	7.0
26-30	213	51.2
>30	174	41.8
**Current marital status**		
Single	55	13.2
Married	361	86.8
**Participant level of Education**		
No formal education	294	70.7

Primary (1-8)	95	22.8
Secondary (9-12)	12	2.9
College and above	15	3.6
**Husband/friend level of education (n=361)**		
No formal education	239	66.21
Primary (1-8)	66	18.28
Secondary (9-12)	43	11.9
College and above	13	3.61
**Read newspaper**		
Yes	47	11.3
No	369	88.7
**How often read a newspaper**		
Always	5	15.1
Once/week	27	64.2
Twice/week	8	9.4
Three and above/week	7	11.3
**Listen to radio?**		
Yes	77	18.5
No	339	81.5
**How often listen radio**		
Always	19	54.8
Once	14	9.1
Twice/week	16	8.1
Three and above/week	28	27.9
**Participant occupation**		
Housewife	305	73.3
Self-employed	19	4.6
Merchant	62	14.9

Private/government employee	30	7.2
**Husband/friend occupation (n= 361)**		
Farmer	280	77.56
Self-employed	70	19.39
Merchant	11	3.05
Private/government employed	16	4.43
**Religion**		
Orthodox	413	99.3
Muslim	3	0.7
**Average monthly income in Ethiopian Birr**		
< 3000	132	54.6
3001-5000	227	31.7
>5000	57	13.7

Note: *physicians

Table 2: Reproductive health service-related characteristics of women in Angolella Tara district of Northeast Ethiopia, 2021 (n= 416)

Characteristics	Frequency	Percentage (%)
**Ever use family planning methods**		
Yes	222	53.4
No	194	46.6
**Pregnancy planned**		
Yes	96	23.1
No	320	76.9
**Have ANC in preceding pregnancy**		
Yes	299	71.9

No	117	28.1
**Know obstetric danger signs**		
Yes	196	47.1
No	220	52.9
**Age at first marriage**		
<18	166	39.9
≥18	250	60.1
**Parity**		
1	60	14.4
2-4	200	48.1
≥5	156	37.5
**Number of live children**		
≤2	115	27.6
3-4	148	35.6
≥5	153	36.8
**History of medical illness**		
Yes	41	9.9
No	375	90.1
**Know the presence of maternal waiting homes**		
Yes	162	38.9
No	254	61.1
**Recent delivery assisted by**		
Skilled healthcare provider	260	62.5
Traditional birth attendant	131	31.5
Family member	25	6.0
**Owns healthcare provider decided by**		
Me only	20	4.8

Husband/partner	16	3.8
Family member	83	20.0
Jointly	297	71.4

ANC=Antenatal care

Table 3: Bi-variable and multivariable logistic regression analysis factors associated with the use of traditional birth attendants by women in Angolella Tara district of Northeast Ethiopia, 2021 (n= 416)

**Variables**	**TBA utilization**		**COR (95%CI)**	**AOR (95%CI)**
	**Yes**	**No**		
**Age in year**				
18-24	4	25	0.26 (.085, 0.77)	0.68 (0.13, 3.64)
25-34	60	153	0.63 (0.41, 0.96)	0.71 (0.62, 2.28)
≥ 35	67	107	1	
**Current marital status**				
Single	20	35	1.29 (0.71, 2.33)	2.63 (1.16, 5.97)*
Married	111	250	1	
**Maternal occupation**				
Housewife	113	192	8.24 (1.93, 35.24)	5.77(0.76, 33.66)
Self-employed	2	17	1.65 (.21, 12.8)	1.24(0.10, 15.05)
Merchant	14	48	4.08 (0.86, 19.3)	10.78(1.0, 60.89)
Private/gov't employed	2	28	1	1
**Age at first marriage**				
<18	79	87	3.46 (2.25, 5.32)	2.31 (1.30, 4.09)*
≥18	52	198	1	
**Pregnancy planned**				
Yes	83	237	0.082 (0.24 0, 0.61)	0.93 (.39, 2.23)
No	48	48	1	
**Have ANC follow-up**				

Yes	47	252	0.07 (0.04, 0.12)	0.11 (.058, 0.21) **
No	84	33	1	
**Number of live children**				
≤2	20	95	0.29 (0.16, 0.51)	0.88 (0.24, 3.26)
3-4	46	102	0.61(0.38, 0.98)	1.55 (0.53, 4.56)
≥5	65	88	1	
**Parity**				
1	9	51	0.24 0.11, .52)	0.43 (0.15, 1.29)
2-4	56	144	0.53 (0.34, 0.83)	1.02 (0.53, 1.97)
≥5	66	90	1	
**Family size**				
<3	6	15	1.0 (0.29, 2.18)	1.63 (0.44, 5.99)
3-5	45	141	1.55 (0.58, 4.16)	1.59 (0.74, 3.43)
>5	80	129	1	
**Listening radio at least once per week**				
Yes	11	66	0.30 (0.16, 0.60)	0.43 (0.18, 0.99) *
No	120	219	1	
**Read newspaper at least once per week**				
Yes	6	41	1	
No	125	244	3.50 (1.45, 8.47)	1.48 (0.14, 1.65)
**Time to reach a health facility**				
< 60 minute	38	204	1	
≥60 minute	93	81	6.16 (3.9, 9.73)	3.46 (1.94, 6.17)**
**Ever use family planning methods**				
Yes	31	191	1	
No	100	94	6.56 (4.09, 10.51)	1.72 (0.82, 3.6)
**Know obstetric danger signs**				
Yes	18	178	1	
No	113	107	10.44 (6.0, 18.14)	5.59 (2.89, 10.81)**

**Ever faced obstetric danger signs**				
Yes	27	168	1	
No	104	117	5.53 (3.41, 8.98)	1.93(0.41, 2.13)
**Assistant for the recent delivery**				
SBA	73	187	1	
TBA	36	95	0.97 (0.61, 1.55)	0.93 (0.49, 1.78)
Family member	22	3	18.79 (5.46, 64.67)	3.52 (0.75, 16.56)
**Know the presence of MWHs**				
Yes	32	130	0.39 (0.24, 0.61)	0.35 (0.18, 1.09)
No	99	155	1	
**Attitude towards TBAs**				
Negative	56	186	1	
Positive	75	99	2.52 (1.65, 3.84)	2.23 (1.24, 4.02) *

Note: *=p-value <0.05, **=p-value <0.001; MWHs = maternity waiting homes; TBAs= traditional birth attendants; SDA= Skilled birth attendant

References

1. WHO/UNFPA/UNICEF. Traditional Birth Attendants: a joint WHO/UNFPA/UNICEF statement. WHO Offset Publ (Geneva). 1992. p. 1–18.
2. Lane K, Garrod J. The return of the Traditional Birth Attendant. J Glob Health [Internet]. 2016;6(2). Available from: 10.7189/jogh.06.020302
3. Choguya NZ. Traditional Birth Attendants and Policy Ambivalence in Zimbabwe. J Anthropol. 2014;2014:1–9.
4. WHO. Strategies toward ending preventable maternal mortality (EPMM). World Heal Organ Geneva, Switz. 2015;
5. World Health Organization (WHO). Maternal mortality. In: World Health Organisation [Internet]. 2019. Available from: https://www.who.int/news-room/fact-sheets/detail/maternal-mortality
6. FMOH. Ethiopian Health Sector Transformation Plan.2015/16 - 2019/20. Vol. 20, FMOH. 2015. 50 p.
7. Shudura E, Yoseph A, Tamiso A. Utilization and Predictors of Maternal Health Care Services among Women of Reproductive Age in Hawassa University Health and Demographic Surveillance System Site, South Ethiopia: A Cross-Sectional Study. Adv Public Heal [Internet]. 2020;2020(5865928):10. Available from: https://doi.org/10.1155/2020/5865928
8. CSA CSA. ETHIOPIA Demographic and Health Survey 2016. CSA, Ethiopia Mini Demographic and Health Survey 2016. Addis Ababa, Ethiopia. 2016. p. 1–551.
9. Ugboaja J, Oguejiofor C, Oranu E, Igwegbe A. Factors associated with the use of traditional birth attendants in Nigeria: A secondary analysis of 2013 Nigeria national demography and health survey. Niger J Gen Pract. 2018;16(2):45.
10. Musa A, Ogunrinde OG, Mamman AI, Saad YM, Ibrahim A, Yakubu AM. Prevalence and pattern of bacterial isolate in febrile children with sickle cell anemia in a tertiary hospital in Northern Nigeria. Sub-Saharan African J Med. 2018;10–4.
11. Miller T, Smith H. Establishing partnership with traditional birth attendants for improved maternal and newborn health: A review of factors influencing implementation. BMC Pregnancy Childbirth. 2017;17(1):1–10.
12. Gurara M, Muyldermans K, Jacquemyn Y, Van geertruyden JP, Draulans V. Traditional birth attendants' roles and homebirth choices in Ethiopia: A qualitative study. Women and Birth [Internet]. 2020;33(5):e464–72. Available from: https://doi.org/10.1016/j.wombi.2019.09.004
13. Ebuehi OM, Akintujoye IA. Perception and utilization of traditional birth attendants by pregnant women attending primary health care clinics in a rural Local Government Area in Ogun State, Nigeria. Int J Womens Health. 2012;4(1):25–34.

14. Ogunyomi MT, Ndikom CM. Perceived Factors Influencing the Utilization of Traditional Birth Attendants ' Services in Akinyele Local Government , Ibadan , Nigeria. J Community Med Prim Heal Care. 2016;28(2):40–8.

15. Adongo P, Atanga R, Yakong V. Demographic characteristics of women that use traditional birth attendants in Bongo District, Ghana. Eur J Midwifery. 2020;4(January):1–6.

16. Atuoye KN, Dixon J, Rishworth A, Galaa SZ, Boamah SA, Luginaah I. Can she make it? Transportation barriers to accessing maternal and child health care services in rural Ghana. BMC Health Serv Res. 2015;15(1):1–10.

17. Serizawa A, Ito K, Algaddal AH, Eltaybe RAM. Cultural perceptions and health behaviors related to safe motherhood among village women in Eastern Sudan: Ethnographic study. Int J Nurs Stud. 2014;51(4):572–81.

18. Sarker BK, Rahman M, Rahman T, Hossain J, Reichenbach L, Mitra DK. Reasons for preference of home delivery with traditional birth attendants (TBAs) in Rural Bangladesh: A qualitative exploration. PLoS One. 2016;11(1):1–19.

19. Institute E public health, ICF. Mini Demographic and Health Survey: key indictors. 2019. 136–148 p.

20. Shirazian T. Around the globe for women's health: a practical guide for the health care provider [Internet]. 2013. 144 p. Available from: 10.1007/978-1-4419-8258-2

21. Ayele GS, Melku AT, Belda SS. Utilization of skilled birth attendant at birth and associated factors among women who gave birth in the last 24 months preceding the survey in Gura Dhamole Woreda, Bale zone, southeast Ethiopia. BMC Public Health [Internet]. 2019;19(1):1–14. Available from: 10.1186/s12889-019-7818-6

22. Mohan D, Lefevre AE, George A, Mpembeni R, Bazant E, Rusibamayila N, et al. Analysis of dropout across the continuum of maternal health care in Tanzania : findings from a cross-sectional household survey. Health Policy Plan [Internet]. 2017;32(March):791–9. Available from: 10.1093/heapol/czx005

23. MoH [Ethiopia]. Obstetrics management protocol for hospitals. 2021. 219 p.

24. Turinawe EB, Rwemisisi JT, Musinguzi LK, De Groot M, Muhangi D, De Vries DH, et al. Traditional birth attendants (TBAs) as potential agents in promoting male involvement in maternity preparedness: Insights from a rural community in Uganda. Reprod Health [Internet]. 2016;13(1):1–11. Available from: http://dx.doi.org/10.1186/s12978-016-0147-7

25. Ogu OMR. Preventing Maternal Mortality during Childbirth: The Scourge of Delivery with Unskilled Birth Attendants. Intech [Internet]. 2016;i:13. Available from: http://dx.doi.org/10.5772/intechopen.90463

26. Akintujoye OMEI. Perception and utilization of traditional birth attendants by pregnant women attending primary health care clinics in a rural Local Government Area in Ogun State , Nigeria. Int J Women's Heal. 2012;4:25–34.

27. Musa A, Ogunrinde OG, Mamman AI, Saad YM, Ibrahim A, Yakubu AM. Prevalence and pattern of bacterial isolate in febrile children with sickle cell anemia in a tertiary hospital in Northern Nigeria: a systematic review. Sub-Saharan African J Med. 2019;6:55–62.

28. Terefe W. Distance from health facility and mothers ' perception of quality related to skilled delivery service utilization in northern Ethiopia. Int J Womens Health. 2017;9:749–56.

29. Yaya S, Bishwajit G, Uthman OA, Amouzou A. Why some women fail to give birth at health facilities : A comparative study between Ethiopia and Nigeria. PLoS One. 2018;13(5):1–11.

30. Kifle MM, Kesete HF, Gaim HT, Angosom GS, Araya MB. Health facility or home delivery ? Factors influencing the choice of delivery place among mothers living in rural communities of Eritrea. J Heal Popul Nutr. 2018;37(22):1–15.

31. Creanga AA, Odhiambo GA, Odera B, Odhiambo O, Desai M, Goodwin M, et al. Pregnant Women ' s Intentions and Subsequent Behaviors Regarding Maternal and Neonatal Service Utilization : Results from a Cohort Study in Nyanza Province , Kenya. PLoS One. 2016;11(9):1–17.

32. Sialubanje C, Massar K, Hamer DH, Ruiter RAC. Personal and environmental predictors of the intention to use maternal healthcare services in Kalomo , Zambia. Health Educ Res [Internet]. 2014;29(6):28–40. Available from: 10.1093/her/cyu057

33. Kadobera D, Sartorius B, Masanja H, Mathew A, Waiswa P. The effect of distance to formal health facility on childhood mortality in rural Tanzania, 2005 Á 2007. 2012;1:1–9.

34. Tanou M, Kamiya Y. Assessing the impact of geographical access to health facilities on maternal healthcare utilization : evidence from the Burkina Faso demographic and health survey 2010. BMC Public Health. 2019;1–8.

35. Kea AZ, Tulloch O, Datiko DG, Theobald S, Kok MC. Exploring barriers to the use of formal maternal health services and priority areas for action in Sidama zone , southern Ethiopia. BMC Pregnancy Childbirth. 2018;18(96):1–12.

36. Mihiretu alemayehu MM. Health care decision making autonomy of women from rural districts of Southern Ethiopia : a community based cross-sectional study. Int J Womens Health. 2017;9:213–21.

37. Dunlop CL, Benova L, Campbell O. Effect of maternal age on facility-based delivery: Analysis of first-order births in 34 countries of sub-Saharan Africa using demographic and health survey data. BMJ Open. 2018;8(4):1–9.

38. Kolola T, Gezahegn T, Addisie M. Health care seeking behavior for common childhood illnesses in jeldu district, oromia regional state, Ethiopia. PLoS One. 2016;11(10):1–11.

39. Amenu G, Mulaw Z, Seyoum T, Bayu H. Knowledge about Danger Signs of Obstetric Complications and Associated Factors among Postnatal Mothers of Mechekel District

Health Centers , East Gojjam Zone , Northwest Ethiopia. Hindawi J Sci. 2016;DOI. https://doi.org/10.1155/2016/3495416.

40. Kebede AA, Cherkos EA, Taye EB. Mother ' s Knowledge of Neonatal Danger Signs and Health-Seeking Practices and Associated Factors in Debretabor , Northwest Ethiopia : A Community-Based Cross-Sectional Study. Res reports Neonatol. 2020;10:47–58.

41. Birmeta K, Dibaba Y, Woldeyohannes D. Determinants of maternal health care utilization in Holeta town , central Ethiopia. MBC Heal Serv Res. 2013;13(256):1–10.

42. Gurara M, Muyldermans K, Jacquemyn Y, Van J, Draulans V. Traditional birth attendants ' roles and homebirth choices in Ethiopia : A qualitative study. Women and Birth [Internet]. 2020;33(5):e464–72. Available from: https://doi.org/10.1016/j.wombi.2019.09.004

43. Vyagusa DB, Mubyazi GM, Masatu M. Involving traditional birth attendants in emergency obstetric care in Tanzania : policy implications of a study of their knowledge and practices in Kigoma Rural District. Int J Equity Health. 2013;12(83):1–14.

44. Temesgen TM, Umer JY, Buda DS, Haregu TN. Contribution of traditional birth attendants to the formal health system in Ethiopia : the case of Afar region. Pan Africa Med J. 2012;13(Supp 1):1–5.

45. Adatara P, Afaya A, Baku EA, Salia SM, Asempah A. Perspective of Traditional Birth Attendants on Their Experiences and Roles in Maternal Health Care in Rural Areas of Northern Ghana. Int J Reprod Med. 2018;17:10.

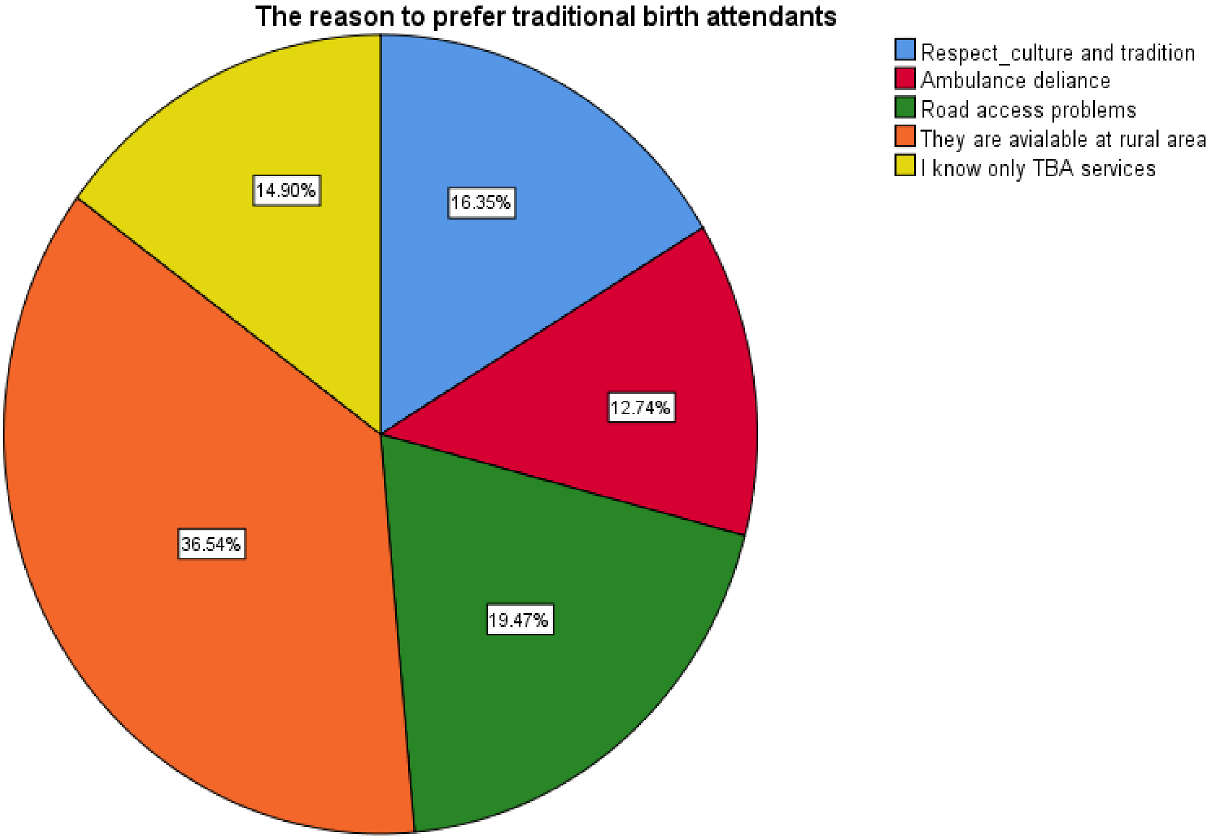

Figure 1. Distribution of reasons for women preference on the use of traditional birth attendants (TBAs) other than skilled birth attendants (SBAs) in Angollela Tata district, northeast Ethiopia 2021

English Version questionaire

Click here to access/download
Supporting Information
S1 file
English version of the questionnaire.docx

SPSS dataset

Click here to access/download
Supporting Information
S2 file
dataset.sav
