# Supplementary material for: Women’s traditional birth attendant utilization at birth and its associated factors in Angolella Tara, Ethiopia
Source: PLoS One. 2022 Nov 11;17(11):e0277504. doi: 10.1371/journal.pone.0277504 (PMC9651568; doi:10.1371/journal.pone.0277504)
Supplement: Attachment — Submitted filename: Response to Reviewers.docx [file pone.0277504.s004.docx]

**September 13, 2022**

**Manuscript PONE-D-22-02742**

**Response to Reviewers**

First of all, we would like to acknowledge your constructive comments on and valuable improvements to our paper. We have incorporated the suggestions and comments in the revised manuscript and clearly show in tracked changes and a point-by-point response, please see below.

**Response to Editor comments**

| S.N | Comments | Responses |
| --- | --- | --- |
| 01 | Please ensure that your manuscript meets PLOS ONE's style requirements, including those for file naming. The PLOS ONE style templates. | Thank you very much for your genuine advice and we have revised the manuscript as per your kind recommendation; PLOS ONE style template. |
| 02 | Please include a caption for figure 1. | Thank you, we have provided at the ed of the manuscript. |
| 03 | Please include captions for your Supporting Information files at the end of your manuscript, and update any in-text citations to match accordingly. | Thank you, we have provided under ‘supporting information heading’. |
| 04 | Please review your reference list to ensure that it is complete and correct. If you have cited papers that have been retracted, please include the rationale for doing so in the manuscript text, or remove these references and replace them with relevant current references. Any changes to the reference list should be mentioned in the rebuttal letter that accompanies your revised manuscript. If you need to cite a retracted article, indicate the article’s retracted status in the References list and also include a citation and full reference for the retraction notice. | Thank you for your kind recommendation. We have added four additional references (i.e. 10, 24, 25 and 27) in the revised version. These references are added to address the reviewers’ comments and during our essential update. |
| 05 | Thank you for submitting the above manuscript to PLOS ONE. During our internal evaluation of the manuscript, we found significant text overlap between your submission and previously published works. Please revise the manuscript to rephrase the duplicated text, cite your sources. | Thank you for pointing out this text overlap. We have revised the manuscript to rephrase the duplicated text, cite sources, and provide details on the advancement of knowledge by this research. |

**Response to Reviewer(s) comments**

**Reviewer #2**

| S.N | Comments | Responses |
| --- | --- | --- |
| 01 | The topic is extremely relevant and is justified by the impact of childbirth care models on maternal mortality. Some questions and suggestions need to be made to further qualify the manuscript.  First, about the third research question: "Why do women prefer TBAs"? In the results, about 31% of the sample used this modality. So, I was wondering why? In the introduction, TBAs are contextualized and presented as a prevalent modality of childbirth care, but why was there no preference for this model in your sample? So the question "Why do women prefer TBAs"? It didn't seem right to me. I suggest modifying or withdrawing this question, and also discussing the results in this perspective. | Thank you for your positive feedback and constrictive comments, we have accepted and modified in the revised manuscript. |
| 02 | n the results, 58.2% have a negative attitude towards TBAs. This percentage is in relation to the total sample or the 131 who used TBAs. | Thank you, the figure (58.2%) is of all study participants. |

**Reviewer #4**

| 01 | Abstract is too long more than 200 words | Thank you for your comment. However, many journals suggest 350, 200 word counts for abstract, PLOS ONE submission guideline allow any length up to 500 words. There are no restrictions on word count, number of figures, or amount of supporting information. The word count for our abstract is 329. |
| --- | --- | --- |
| 02 | utilization and prevalence are not suitable as keywords | You have pointed out an important issue. We have corrected. |
| 03 | Methods: there is no validity and reliability test for instrument??? | Thank you, we have incorporated in the revised manuscript. |
| 04 | Ethical consideration: should write the letter number of ethical approval | Thank you very much, provided. |
| 05 | Result: table 3 is not complete, no p value in each varibles and as logistic regression analyzis it needs to display the candidates that selected, then the multi variate analyzis of the selected candidates, end of the result should be the most influenced factor toward the dependent variable. | Thank you, dear reviewer, we have provided it. However, p-values simply provide a cut-off beyond which we assert that the findings are 'statistically significant' (by convention, this is p<0.05). Confidence intervals provide information about statistical significance, as well as the direction and strength of the effect. |
| 06 | Discussion: 2nd sentence need reference. | Comment accepted and corrected. |
| 07 | Discussion: page 9, 1st paragraph: not clear statement (Nigerian government or Ethiopia government because the result in this study is better than in Nigeria (31,5% versus 65%) | Thank you, the Nigerian government encourage TBAs service integration with skilled birth attendants for childbirth. As a result, 65% TBA utilization at birth. Ethiopian gov’t is better (31.5% TBA utilization). |
| 08 | page 9: last sentence: this rationalization is not in accordance with the research results above. | Really thank you for your effort. Corrected. Please kindly see the tracked changes. |
| 09 | page 10: 2nd paragraph: The rationalization does not fit in the explanation here but is more appropriate in explaining the results that women who are pregnant for the first-time use TBA services more. | Your kind suggestion is accepted. |
| 10 | Conclusion: Content is not enough conclusion, did not answer the objectives of the study, most of statement and paragraph is the conclusion are recommendation. | Thank you, all relevant findings incorporated. |
| 11 | please revise table 3: no p value in each variable?? it needs one column for p (significant value). Not clear multi variate analyzis result / logistic regression (in the table should display the p value in each variable, follow by choosing the candidate for multy analyzis etc). | Comment accepted and corrected. |
| 12 | please check the references list some references have no publisher???URL?? when cited? | Thank you, some references amend accordingly. |

**Reviewer #5**

**(Dr. Vanice Ferrazza Zaltron)**

| 01 | Please provide a grammatical native speaker review. | Thank you for the comment, the language was reviewed exhaustively. It is also clearly visualized via track changes in the revised manuscript. |
| --- | --- | --- |
| 02 | Introduction: Please provide a better writting to a better understanding; Ex.  "Although women believed that that complication might arise from TBA care (13), low maternal educational level, rural residence, poor family wealth index, unemployed status, having >5 living children, married, predominantly farmers, married to spouses who were farmers without formal education, user-friendly care from TBAs, easily  accessible, delivery environment familiar to the clients’ respect from TBAs for the religious beliefs of clients were significant factors for women to used TBAs." It is too long and of difficult comprehension. | Thank you for your deep review, paraphrased. |
| 03 | Methods: Please provide inclusion and exclusion criteria more clearly. | Thank you, provided. |
| 04 | Discussion is adequate, but I suggest to the authors to improve the strenghts information in this section (limitations of the study). I miss information about improving educational information. | Thank you, provided. |
